# Unaltered Prion Pathogenesis in a Mouse Model of High-Fat Diet-Induced Insulin Resistance

**DOI:** 10.1371/journal.pone.0144983

**Published:** 2015-12-14

**Authors:** Caihong Zhu, Petra Schwarz, Irina Abakumova, Adriano Aguzzi

**Affiliations:** Institute of Neuropathology, University Hospital Zurich, Zurich, Switzerland; Van Andel Institute, UNITED STATES

## Abstract

Epidemiological, clinical, and experimental animal studies suggest a strong correlation between insulin resistance and Alzheimer’s disease. In fact, type-2 diabetes is considered an important risk factor of developing Alzheimer’s disease. In addition, impaired insulin signaling in the Alzheimer’s disease brain may promote Aβ production, impair Aβ clearance and induce tau hyperphosphorylation, thereby leading to deterioration of the disease. The pathological prion protein, PrP^Sc^, deposits in the form of extracellular aggregates and leads to dementia, raising the question as to whether prion pathogenesis may also be affected by insulin resistance. We therefore established high-fat diet-induced insulin resistance in *tg*a*20* mice, which overexpress the prion protein. We then inoculated the insulin-resistant mice with prions. We found that insulin resistance in *tg*a*20* mice did not affect prion disease progression, PrP^Sc^ deposition, astrogliosis or microglial activation, and had no effect on survival. Our study demonstrates that in a mouse model, insulin resistance does not significantly contribute to prion pathogenesis.

## Introduction

Type-2 Diabetes (T2D) represents one of the top threats to human health, and currently afflicts hundreds of millions of people worldwide (http://www.idf.org/diabetesatlas/update-2014). Diet-induced obesity is thought to be the primary cause of T2D, and insulin resistance is considered the central clinical characteristic of what has been called the "metabolic syndrome" [1]. Impaired insulin responsiveness in fat, muscle, and liver cells leads to failure of glucose absorption despite excessive insulin secretion, resulting in hyperglycemia and associated organ damage in T2D patients.

Intriguingly, T2D is also associated with changes in learning, memory, cognitive flexibility and processing speed [2]. Peripheral insulin resistance has been reported to go along with impaired glucose metabolism in the brain [3–6]. Unequivocal evidence from epidemiological studies indicates that T2D and insulin resistance are linked to an increased risk of sporadic Alzheimer’s disease (AD), the most prevalent form of dementia affecting the elderly [7–12]. In addition, impaired insulin signaling in the brain has been observed in both AD patients and animal models of AD [13–20]. However, the molecular mechanisms underlying the connection between insulin resistance and dementia are far from understood.

Extensive studies have been carried out in animal models to delineate the role of insulin resistance in AD pathogenesis. High-fat diet-induced dysfunction of insulin signaling in rats reduces expression of brain-derived neurotrophic factor (BDNF) and leads to cognitive impairment [21]. Wild-type C57BL/6 mice fed with high-fat diet develop insulin resistance in the brain, associated with increased tau phosphorylation, reduced post-synaptic protein PSD95 and cognitive impairment [22, 23]. Additional mouse models have suggested that defective brain insulin signaling can accelerate or exacerbate AD pathology by increasing Aβ deposition (through promotion of its production and/or impairment of its clearance) and inducing tau hyperphosphorylation, [24–30]. In cynomolgus monkeys, T2D accelerates Aβ pathology by exacerbating endocytic malfunction [31]. Conversely, Aβ oligomers that accumulate in AD brains have been reported to inhibit autophosphorylation of insulin receptor, reduce insulin receptors on plasma membranes, and decrease insulin signaling in neuronal cells [32, 33]. Furthermore, cerebrally derived Aβ can be transported and accumulated in pancreatic β-cells, thereby triggering islet degeneration and impairing insulin production [28]. Accordingly, the interaction between AD and insulin resistance may be reciprocal and bidirectional.

Prion diseases are a group of fatal neurodegenerative disorders comprising various veterinary diseases (scrapie, bovine spongiform encephalopathy, chronic wasting disease), as well as Creutzfeldt-Jakob disease, kuru, Gerstmann-Sträussler-Scheinker disease and fatal familiar insomnia in humans [34]. Prion diseases are characterized by the extracellular deposition of PrP^Sc^, an abnormal isoform of the cellular prion protein (PrP^C^) [35]. Many observations suggest a possible association between insulin signaling and prion disease. For one, insulin treatment enhances PrP^C^ expression in neuronal cell lines, and PrP deficiency results in insulin signaling impairment and glucose-intolerance [36–38]. Moreover, aberrant insulin receptor processing and function was observed in prion-infected neuroblastoma cells [39, 40]. Recently, it has been reported that prion infection also reduces cell surface expression of insulin receptor and weakens insulin signaling in neuronal cells [41]. Finally, in an animal model of high-fructose diet-induced insulin resistance, PrP^C^ expression is downregulated [42].

The above studies are tantalizing, but they do not prove a cross-talk between impaired glucose metabolism and the pathogenesis of prion disease. Therefore, we examined the effect of high-fat diet-induced insulin resistance on prion pathogenesis. We first established insulin resistance by feeding mice with a high-fat diet for 4 weeks followed by intracerebral inoculation with prions. Prion pathogenesis in mice suffering from high-fat diet-induced insulin resistance was indistinguishable from that of control mice on a normal diet in all investigated parameters, suggesting that insulin resistance does not significantly affect prion pathogenesis.

## Material and Methods

### Animals & ethical statement

Female *tg*a*20* mice were maintained in high hygienic grade facility and housed 3 mice per cage under a 12 h light/12 h dark cycle (from 7 am to 7 pm) at 21±1°C, and fed with either high fat diet (Research Diets, #D12492) or normal diet and water *ad libitum*. HFD were changed twice a week to avoid oily fur or bedding. Body weights were measured weekly. All animal experiments were carried out in strict accordance with the Rules and Regulations for the Protection of Animal Rights (Tierschutzgesetz and Tierschutzverordnung) of the Swiss Bundesamt für Lebensmittelsicherheit und Veterinärwesen and were preemptively approved by the Animal Welfare Committee of the Canton of Zürich (permit # 41/2012).

### Fasting blood glucose and plasma insulin measurement

Mice were first fasted for 6 hours. Before retro-orbital bleeding, mice were anesthetized with isoflurane. Blood glucose levels were measured by Accu-Chek Aviva test stripes. To test plasma insulin level, plasma were prepared from the blood and a Rat/Mouse Insulin ELISA kit (Millipore AG, EZRMI-13K) was used to determine the plasma insulin level according to the manufacturer’s instruction.

### Glucose and insulin tolerance tests

Glucose and insulin tolerance tests were performed according to published guidelines[43]. To test intraperitoneal glucose tolerance, *tg*a*20* mice were first fasted for 6 hours. Mice were then intraperitoneally injected with glucose 1g/kg (Sigma). Blood glucose levels were measured from the tail vein at 0, 15, 30, 45, 60 and 120 min after glucose injection. Insulin tolerance tests were performed similarly with a dose 1 IU/kg of human insulin (Sigma). Blood glucose levels from the tail vein were measured at 0, 15, 30, 45, 60 and 120 min after insulin injection.

### RML6 inoculation

Mice were inoculated intracerebrally (i.c) with 30 μl of brain homogenate diluted in PBS with 5% BSA and containing 3 x 5 log LD50 units of the Rocky Mountain Laboratories scrapie strain (passage 6, thus called RML6). Mice were monitored and actions were taken to minimize animal suffering and distress according to the details described in S1 Table. Scrapie was diagnosed according to clinical criteria (ataxia, limb weakness, front leg paresis and rolling). Mice were sacrificed by CO_2_ inhalation on the day of appearance of terminal clinical signs of scrapie (specific criteria in S1 Table), organs were taken and then were either snap-frozen for biochemical analysis or fixed in 4% formalin for histological assessment. The time elapsed from prion inoculation to the terminal stage of disease was defined as incubation time for the survival study.

### Quantitative real-time PCR (qRT-PCR)

Total RNA from each brain was extracted using TRIzol (Invitrogen) according to the manufacturer’s instruction. The quality of RNA was analyzed by Bioanalyzer 2100 (Agilent Technologies), RNAs with RIN>7 were used for cDNA synthesis. cDNA were synthesized from ~1 μg total RNA using QuantiTect Reverse Transcription kit (QIAGEN) according to the manufacturer’s instruction. Quantitative real-time PCR (qRT-PCR) was performed using the SYBR Green PCR Master Mix (Roche) on a ViiA7 Real-Time PCR system (Applied Biosystems). Expression levels were normalized using Gapdh. The sequence of qRT-PCR primers are: *Gapdh* sense, 5´-CCA CCC CAG CAA GGA GAC T-3´;antisense, 5´-GAA ATT GTG AGG GAG ATG CT-3´. *Prnp* sense, 5´-GCC AGT GGA TCA GTA CAG CA-3´; antisense, 5´-ATC CCA CGA TCA GGA AGA TG-3´. *TNFα* sense, 5´-ACT TCG GGG TGA TCG GTC CCC-3´; antisense, 5´-TGG TTT GCT ACG ACG TGG GCT AC-3´. *IL-6* sense, 5´-TCC AAT GCT CTC CTA ACA GAT AAG-3´; antisense, 5´-CAA GAT GAA TTG GAT GGT CTT G-3´.; *IL-1β* sense, 5´-TGC AGC TGG AGA GTG TGG ATC CC-3´; antisense, 5´-TGT GCT CTG CTT GTG AGG TGC TG-3´.

### Western blot analysis

To detect PrP^C^ in the mouse brains, one hemisphere of each brain was homogenized with buffer PBS containing 0.5% Nonidet P-40 and 0.5%CHAPSO. Total protein concentration was determined using the bicinchoninic acid assay (Pierce). ~8 ug proteins were loaded and separated on a 12% Bis-Tris polyacrylamide gel (NuPAGE, Invitrogen) and then blotted onto a nitrocellulose membrane. Membranes were blocked with 5% wt/vol Topblock (LuBioScience) in PBS supplemented with 0.1% Tween 20 (vol/vol) and incubated with primary antibodies POM1 in 1% Topblock (400 ng ml^−1^) overnight. After washing, the membranes were then incubated with secondary antibody horseradish peroxidase (HRP)-conjugated rabbit anti–mouse IgG1 (1:10,000, Zymed). Blots were developed using Luminata Crescendo Western HRP substrate (Millipore) and visualized using the Stella system (model 3000, Bio-Rad). To avoid variation in loading, the same blots were striped and incubated with anti-actin antibody (1:10,000, Millipore). The PrP^C^ signals were normalized to actin as a loading control. Similar protocol was used for Western blot to detect p^Ser473^-Akt and total Akt, p^Ser9^-GSK3β and total GSK3β. The primary antibodies used were Phospho-Akt (Ser473) (D9E) XP® Rabbit mAb (Cell Signaling Technology, 4060), Akt (pan) (11E7) Rabbit mAb (Cell Signaling Technology, 4685), Phospho-GSK-3-beta (Ser9) (D3A4) Rabbit mAb (Cell Signaling Technology, 9322), GSK3β (27C10) Rabbit mAb (Cell Signaling Technology, 9315). The secondary antibody used was HRP-conjugated goat anti–rabbit IgG1 (1:10,000, Jackson ImmunoResearch, 115-035-045)

To detect PrP^Sc^, prion infected forebrains were homogenized in sterile 0.32 M sucrose in PBS. Total protein concentration was determined using the bicinchoninic acid assay (Pierce). Samples were adjusted to 20 μg protein in 20 μl and digested with 50 μg ml^−1^ proteinase K in digestion buffer (PBS containing 0.5% wt/vol sodium deoxycholate and 0.5% vol/vol Nonidet P-40) for 45 min at 37°C. PK digestion was stopped by adding loading buffer (Invitrogen) and boiling samples at 95°C for 5 min. Proteins were then separated on a 12% Bis-Tris polyacrylamide gel (NuPAGE, Invitrogen) and blotted onto a nitrocellulose membrane. POM1 and horseradish peroxidase (HRP)-conjugated rabbit anti–mouse IgG1 were used as primary and secondary antibodies, respectively. Blots were developed using Luminata Crescendo Western HRP substrate (Millipore) and visualized using the FUJIFILM LAS-3000 system. To detect IBA-1 and GFAP in prion-infected brains by Western blot, 20 μg of total brain protein were loaded and anti-IBA-1 antibody (1:1000; Wako Chemicals GmbH, Germany) anti-GFAP antibody (D1F4Q) XP® Rabbit mAb (1:3000; Cell Signaling Technology, 12389) were used. Actin was used as loading control.

### Enzyme-linked Immunosorbent Assay (ELISA)

PrP^C^ in ND- or HFD- fed mouse brains was quantified by sandwich ELISA as described previously[44]. Briefly, 96-well plates were coated with 400ng/ml of purified POM1 antibodies overnight at 4°C. Plates were washed with PBS containing 0.1% (vol/vol) Tween 20 (PBST), and blocked with 5% TopBlock for 2hrs at room temperature. After washing, plates were incubated with 50 ul of serially diluted samples in PBST containing 1% TopBlock, starting from a 50ug/ml dilution. After 2hrs at room temperature, plates were washed extensively and then probed with biotinylated POM2 at a concentration of 200ng/ml in PBST containing 1% TopBlock for 1hr at room temperature. Subsequently, plates were washed and incubated with horseradish peroxidase conjugated Avidin (1:1000 dilution, Pharmingen No. 554058) for 1hr at room temperature. Plates were developed with TMB (3,3', 5,5;-tetramethylbenzidine) for 15–30min and stopped by adding 0.5M H_2_SO_4_, and optical density was measured at 405nm. Titer was defined as the highest dilution showing an OD more than two times the technical background, which was calculated as the average of uncoated wells.

### Immunohistochemistry

Formalin-fixed tissues were treated with concentrated formic acid for 60 min to inactivate prion infectivity and embedded in paraffin. Paraffin sections (2mm) of brains were stained with hematoxylin/eosin (HE). After deparaffinization through graded alcohols, antibodies GFAP (1:300; DAKO, Carpinteris, CA) for astrocytes were applied and visualized using standard methods, IBA-1 (1:1000; Wako Chemicals GmbH, Germany) was used for highlighting activated microglial cells. Stainings were visualized using DAB (Sigma-Aldrich), with a hematoxylin counterstain applied subsequently. For partially protease K-resistant prion protein deposition staining, deparaffinized sections were incubated for 6 min in 98% formic acid and washed in distilled water for 30 min. Sections were incubated in Ventana buffer and stains were performed on a NEXEX immunohistochemistry robot (Ventana instruments, Switzerland) using an IVIEW DAB Detection Kit (Ventana). After incubation with protease 1 (Ventana) for 16 min, sections were incubated with anti-PrP SAF-84 (SPI bio, A03208, 1:200) for 32 min. Sections were counterstained with hematoxylin. Sections were imaged using a Zeiss Axiophot light microscope.

### Statistical analysis

Results are presented as the mean of replicas ± standard error of the mean (SEM). Statistical significance between experimental groups was assessed using an unpaired Student’s t-Test or two-way analysis of variance (ANOVA). Incubation times were analyzed using the Kaplan-Meier method and compared between groups using the logrank test. *p*-values <0.05 were considered statistically significant.

## Results

### High-fat diet-induced insulin resistance in tga20 mice

**Fig 1:**
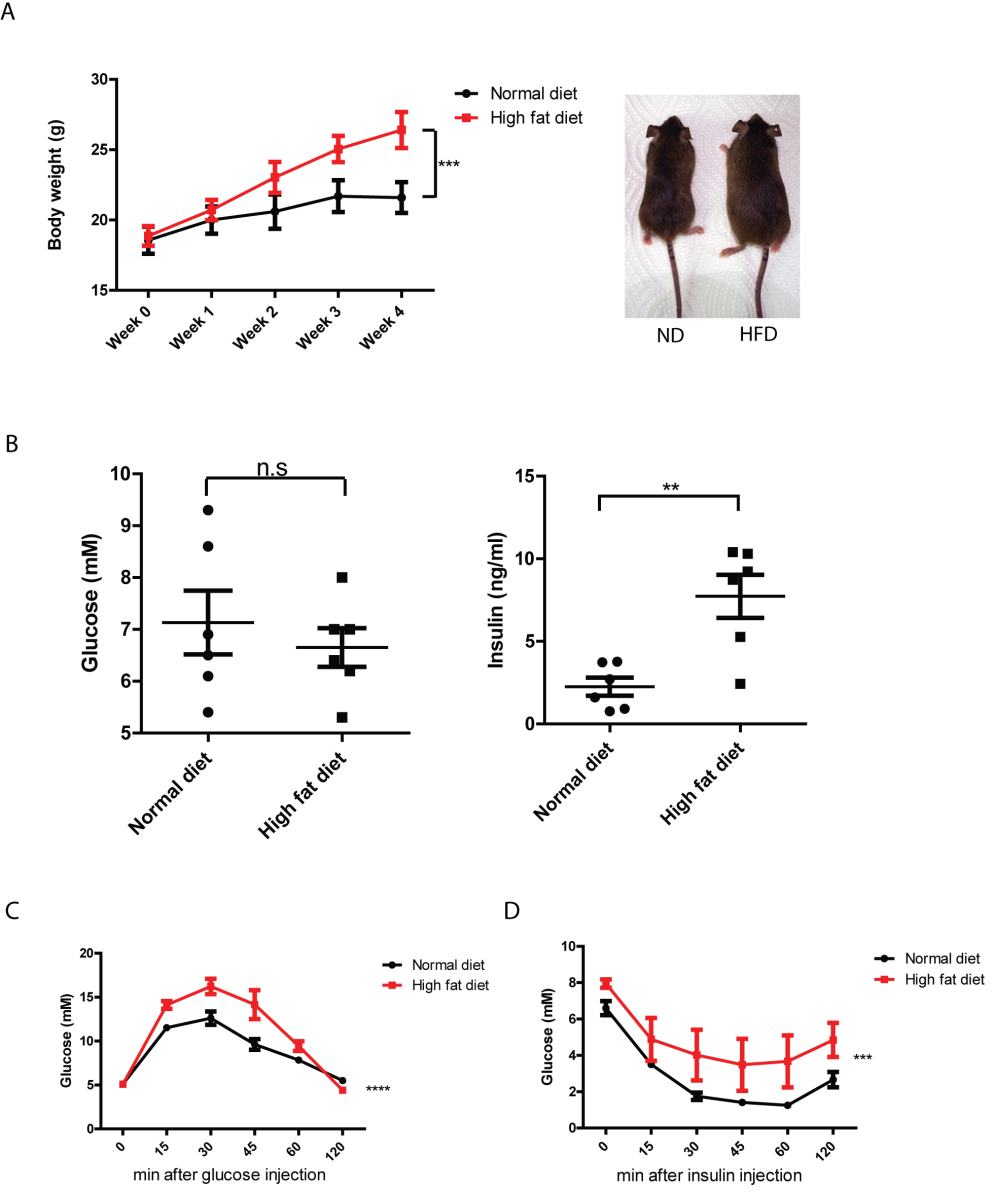
High-fat diet induced insulin resistance in *tg*a*20* mice. (**A**) Left: Body weight of *tg*a*20* mice fed with either high-fat diet (HFD) or normal diet (ND). After 4 weeks, HFD-fed mice gained significantly more body weight than ND-fed mice after 4 weeks. (n = 6; ***: *p*<0.001). Right: appearance of mice fed with ND or HFD for 4 weeks. (**B**) Fasting glucose and insulin level in HFD-fed and ND-fed mice. HFD-fed mice had similar levels of fasting glucose, but showed significantly higher fasting insulin levels. (n = 6; n.s: *p*>0.05; **: *p*<0.01). (**C**) glucose tolerance test and (**D**) insulin tolerance test of HFD-fed and ND-fed mice. HFD-fed mice showed impaired response to intraperitoneally injected glucose and insulin (n = 6; ***: *p*<0.001; ****: p<0.0001).

High-fat diet (HFD) induced insulin resistance has been well-established in various inbred mouse strains [45–48]. The success of insulin resistance induction by diet is highly dependent on the mouse genetic background [46, 47]. In order to induce insulin resistance in *tg*a*20* mice, a transgenic line that overexpress prion protein and was generated on a 129Sv x C57BL/6 genetic background [49], 6–8 weeks old female *tg*a*20* mice were fed with either a HFD containing 60% kcal from fat, or with a normal diet (ND) consisting of 10% kcal from fat (n = 6 per group). Body weight was measured weekly. After 4 weeks, the HFD treated group showed significantly higher body weights than ND group, and HFD fed mice show obesity (Fig 1A). Fasting blood glucose and plasma insulin levels were also compared between HFD and ND groups. While fasting glucose level was not obviously different between these two groups, fasting insulin level was significantly increased in HFD group (Fig 1B), indicating that hyperinsulinemia had been attained in *tg*a*20* mice after 4 weeks of treatment.

We next performed intraperitoneal glucose and insulin tolerance tests on *tg*a*20* mice after 4 weeks of a HFD or ND. The HFD-treated *tg*a*20* mice showed impaired glucose and insulin tolerance compared to mice fed with a ND (Fig 1C and 1D). Therefore, 4 weeks of HFD feeding is sufficient to induce insulin resistance in *tg*a*20* mice. Taken together, these data indicate that HFD treatment on *tg*a*20* mice for 4 weeks resulted in obesity, hyperinsulinemia and insulin resistance.

### Dysregulated insulin signaling but unchanged PrP^C^ expression in the brains of tga20 mice on a high-fat diet

**Fig 2:**
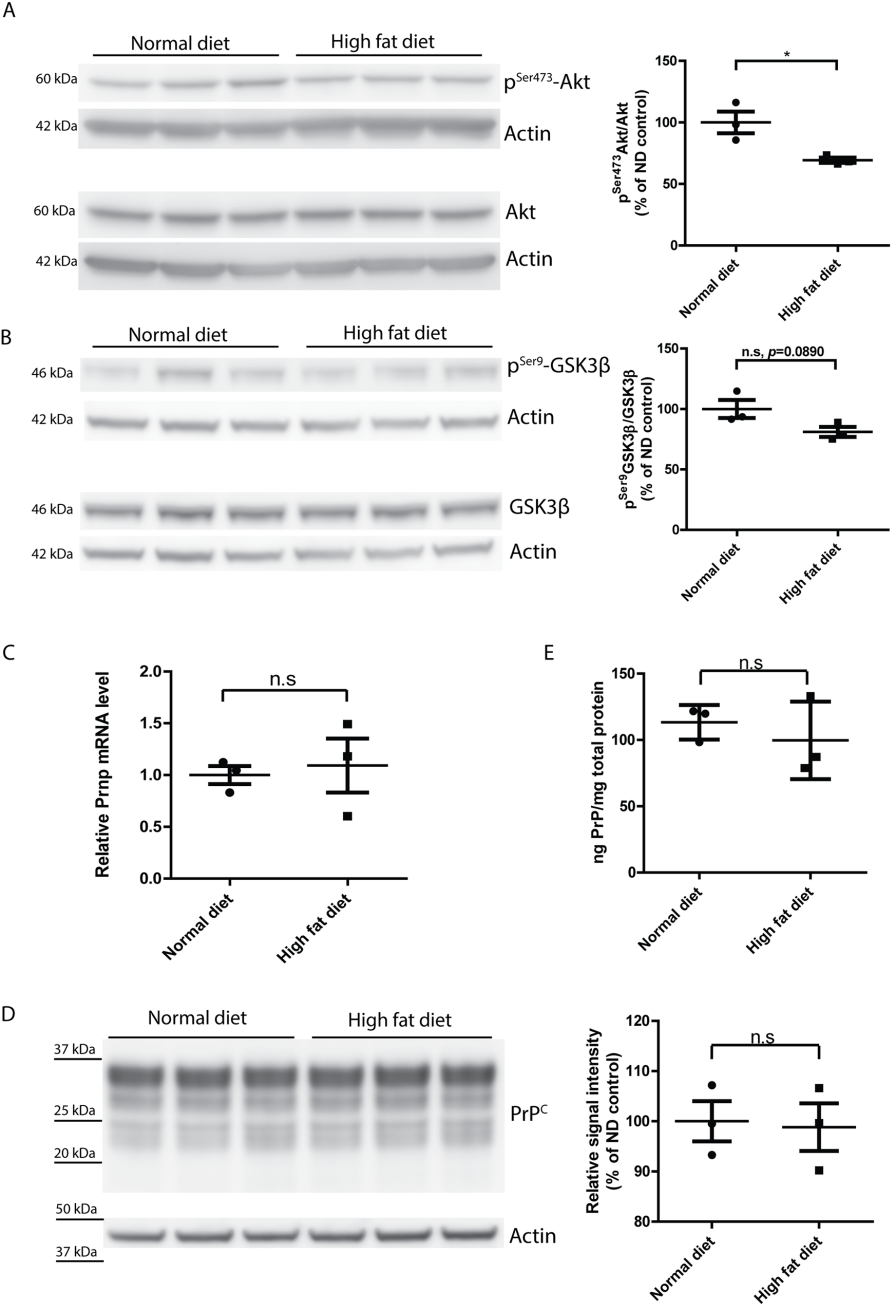
Dysregulated insulin signaling but unchanged PrP^C^ expression in HFD-fed mouse brains. (**A**) Left: Western blot of p^Ser473^-Akt and total Akt in ND- or HFD-fed mouse brains. Right: densitometric quantification of the Western blot showed significantly lower p^Ser473^-Akt/Akt ratio in HFD-fed mouse brains (n = 3, *, *p* = 0.0280) (**B**) Left: Western blot of p^Ser9^-GSK3β and total GSK3β in ND- or HFD-fed mouse brains. Right: densitometric quantification of the Western blot revealed a trend of reduced lower p^Ser9^-GSK3β/ GSK3β ratio in HFD-fed mouse brains (n = 3, n.s *p* = 0.0890). (**C**) qRT-PCR of *Prnp* expression in HFD-fed and ND-fed mouse brains showed similar levels of *Prnp* mRNA (n = 3, n.s *p*>0.05). (**D**) Left: Western blot of PrP^C^ in HFD-fed and ND-fed mouse brains. Right: densitometric quantification of the Western blot demonstrated a similar level of PrP^C^ expression (n = 3, n.s *p*>0.05). **(E)** ELISA of PrP^C^ showed similar level of PrP^C^ expression in HFD-fed and ND-fed mouse brains (n = 3, n.s *p*>0.05).

Peripheral insulin resistance is often associated with impaired insulin signaling and dysregulated glucose metabolism in the brain [3–6]. We therefore sought to determine whether HFD feeding led to dysregulation of insulin signaling in *tg*a*20* mouse brain. We first assessed the levels of Ser^473^-phosphorylated Akt (p^Ser473^-Akt) which is downstream of insulin signaling and phosphorylated by PI3K. We found that the level of p^Ser473^-Akt was significantly lower (*, *p* = 0.028) in HFD group, suggesting an impairment of insulin signaling in HFD brains (Fig 2A and S1 Fig). We also measured the level of p^Ser9^-GSK3β, a form of GSK3β phosphorylated by activated Akt. We observed a trend of reduced level of p^Ser9^-GSK3β (n.s, *p* = 0.0890) in the HFD group (Fig 2B and S2 Fig), suggesting that insulin signaling in HFD fed mouse brain was indeed impaired.

The level of cellular prion protein (PrP^C^) is the main determinant of the speed of prion pathogenesis. A 50% reduction of PrP^C^ expression, e.g. brought about by hemizygosity in the *Prnp* gene, conspicuously prolongs prion incubation time [50]. Conversely, overexpression of PrP^C^ remarkably accelerates prion progression [49]. It was reported that high-fructose diet-induced insulin resistance reduces PrP^C^ expression in rats [42]. We therefore assessed whether HFD treatment affects PrP^C^ expression. However, qRT-PCR for *Prnp* transcripts, Western blot and ELISA for PrP^C^ altogether showed that PrP^C^ expression was not changed by HFD treatment (Fig 2C–2E).

### Unaltered prion pathogenesis in insulin resistant tga20 mice

**Fig 3:**
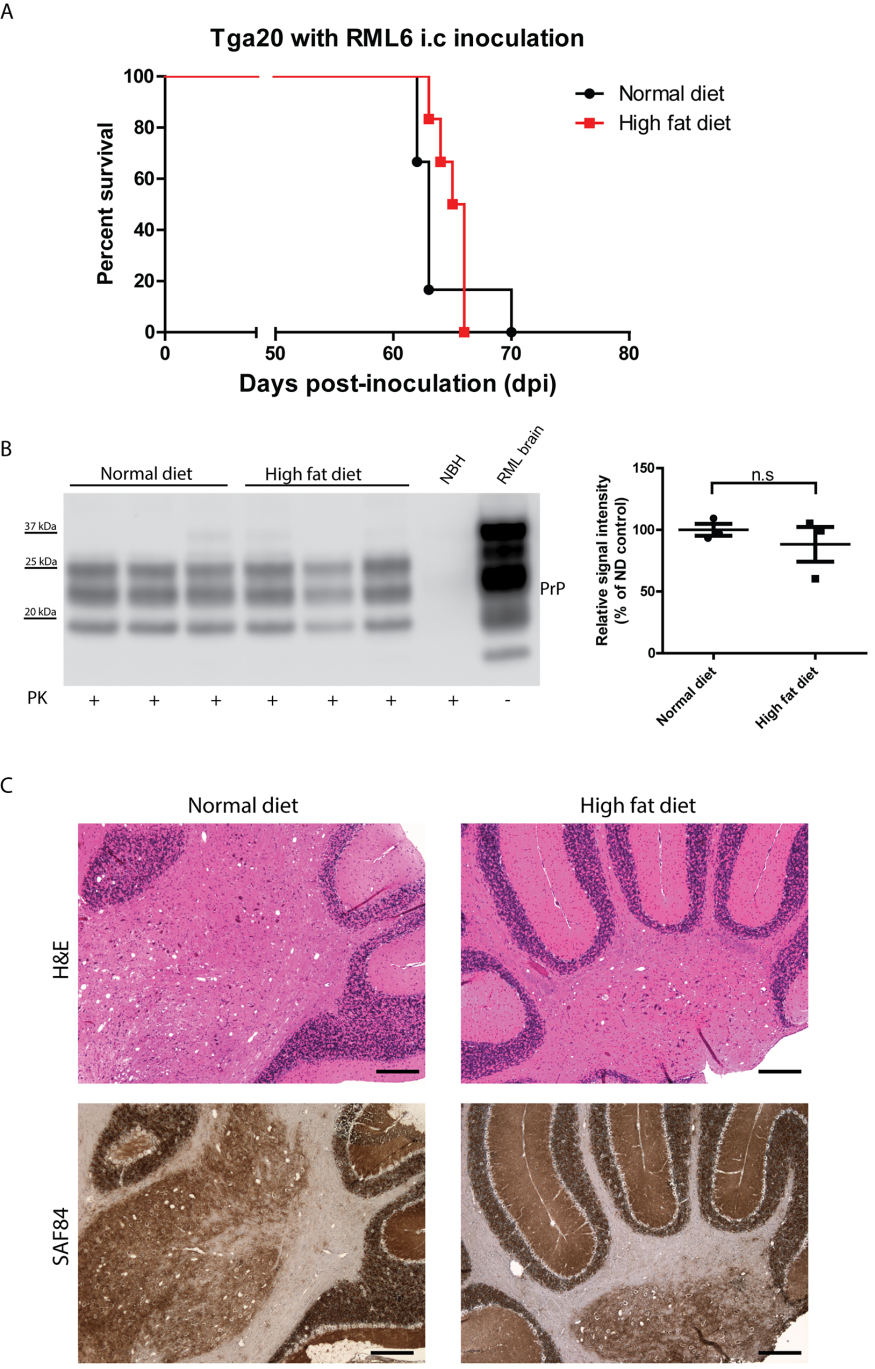
Unaltered prion pathogenesis in HFD-fed *tg*a*20* mice. (**A**) Survival curve of HFD-fed and ND-fed *tg*a*20* mice intracerebrally inoculated with RML6. There was no significant difference in survival between the two groups (n = 6 per group; n.s.: *p*>0.05). (**B**) Left: Western blot for proteinase K resistant PrP^Sc^ in terminally sick mouse brains. Right: densitometric quantification of the Western blot revealed no significant difference between HFD-fed and ND-fed *tg*a*20* mouse brains (n = 3, n.s *p*>0.05). (**C**) Representative histology of terminally sick HFD-fed and ND-fed *tg*a*20* mouse brains stained for H&E and SAF84. There was no obvious difference between the two groups in lesion pattern, vacuolation, and PrP^Sc^ deposition. Scale bar = 200μm.

Having established insulin resistance in *tg*a*20* mice, we further evaluated the effect of insulin resistance on prion pathogenesis. *Tg*a*20* mice were first fed with HFD for 4 weeks to induce insulin resistance. Next, HFD-fed or ND-fed control *tg*a*20* mice were inoculated intracerebrally (i.c) with RML6. The ND vs. HFD feeding was maintained in the respective groups throughout the entire experimental period. The incubation time was defined as the time elapsed from prion inoculation to the terminal stage of disease (the day on which mice showed severe scrapie symptoms requiring euthanasia). We found that both HFD and ND fed groups succumbed to prion disease with similar incubation times (ND: median survival = 63 dpi, HFD: median survival = 65.5 dpi; *p* = 0.307) (Fig 3A), suggesting that HFD-induced insulin resistance does not significantly contribute to the prion progression. No unexpected death was observed in this study.

Next, we assessed proteinase K-resistant PrP^Sc^ deposition in terminally sick mouse brains. Western blots revealed that there were similar PrP^Sc^ levels in the HFD and ND groups (Fig 3B). Histological analysis of terminally sick mouse brains also demonstrated that HFD treatment does not alter prion pathology, as spongiform changes were qualitatively similar in the two groups (Fig 3C).

**Fig 4:**
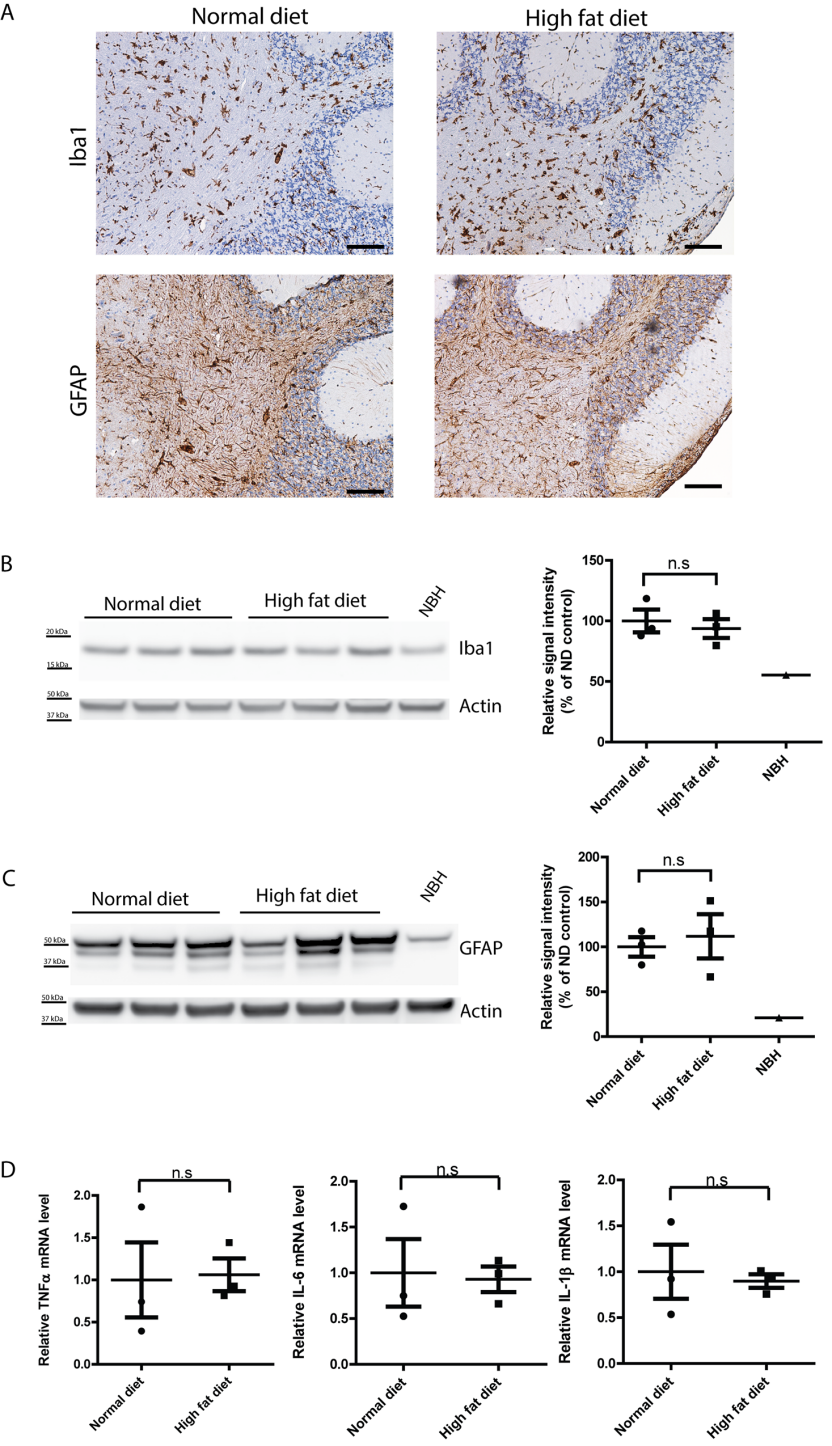
Similar level of astrogliosis and microglial activation in terminally sick HFD- and ND-fed *tg*a*20* mice. (**A**) Representative immunohistochemical staining for GFAP and Iba1 in brains of terminally sick HFD-fed and ND-fed *tg*a*20* mice. There was no detectable difference between the two groups in astrogliosis or microglial activation. Scale bar = 100μm. (**B**) Left: Western blot for Iba1 in terminally sick mouse brains. Right: densitometric quantification of the Western blot revealed no significant difference of Iba1 levels between HFD-fed and ND-fed *tg*a*20* mouse brains (n = 3; n.s.: *p*>0.05). (**C**) Left: Western blot for GFAP in terminally sick mouse brains. Right: densitometric quantification of the Western blot showed no significant difference of GFAP levels between HFD-fed and ND-fed *tg*a*20* mouse brains (n = 3; n.s.: *p*>0.05). (**D**) qRT-PCR of cytokines TNFα, IL-6 and IL-1β expression revealed similar expression levels of these cytokines in terminally sick HFD-fed and ND-fed mouse brains (n = 3; n.s.: *p*>0.05).

Astrogliosis and microglial activation are both associated with prion pathogenesis. Immunohistochemical staining of the astrocyte marker GFAP and microglial marker Iba1 showed similar levels of astrogliosis and microglial activation in terminally sick mice fed witht HFD or ND (Fig 4A). The latter findings were further confirmed by Western blot of GFAP and Iba1 for brains from terminally sick HFD- and ND-fed mice (Fig 4B and 4C). Cytokine profiling of terminally sick mouse brains also failed to reveal an obvious difference between HFD and ND groups (Fig 4D). Overall, we conclude that insulin resistance does not alter prion progression, PrP^Sc^ deposition, astrogliosis and microglial activation.

## Discussion

Increasing evidence from epidemiological and clinical studies suggests that impaired insulin signaling is linked to neurodegeneration [7–12]. Specifically, reduced insulin level and insulin activity may contribute to the pathological processes associated with AD. Experimental studies using mouse models of AD and diet-induced insulin resistance suggest several molecular pathways by which insulin resistance may aggravate AD pathology [24–30]. Therefore, supplementation of insulin to the brain may provide therapeutic potential benefits to AD patients. Indeed, intranasal administration of insulin to AD patients has been shown to modulate plasma Aβ levels, enhance memory and improve cognition [51–55]. These encouraging results prompted us to explore whether insulin administration could also benefit other neurodegenerative diseases. We therefore aimed to determine the effect of insulin resistance in prion pathogenesis.

We first introduced a high-fat diet regime, which is well-established and widely used to induce insulin resistance in rodents, to *tg*a*20* mice, a transgenic mouse line overexpressing PrP^C^ that reliably experiences prion pathogenesis with rapid progression upon infection. Four weeks of high-fat diet is sufficient to induce insulin resistance, as evidenced by obesity, hyperinsulinemia and impaired glucose and insulin tolerance. The extent of insulin resistance in the model established here was not qualitatively different from other models using wild-type mice [22, 23]. In line with previous reports [24–30], HFD-induced insulin resistance was also associated with impaired insulin signaling in the brain. We then inoculated the insulin resistant *tg*a*20* mice with prions. Interestingly, high-fat diet-fed *tg*a*20* mice displayed similar prion progression and pathogenesis to that of normal diet-fed *tg*a*20* mice, suggesting insulin resistance does not alter prion-induced neurotoxicity, PrP^Sc^ deposition and neuroinflammation. The absence of an effect of insulin resistance on prion pathogenesis also indicates that distinct cellular and molecular pathways are employed by PrP^Sc^ and Aβ for accumulation and clearance.

The use of *tg*a*20* transgenic mice may be viewed as a possible limitation of the present study. These mice ovexpress the prion protein and experience a much more rapid pathogenesis of scrapie than wild-type mice. However, it is conceivable that the natural course of scrapie in *tg*a*20* mice may differ from that of wild-type mice. We deem the use of *tg*a*20* mice justifiable and informative in this context for several reasons. Firstly, a wealth of studies with *tg*a*20* mice has failed to uncover any relevant deviation from wild-type mice in pathogenesis, except for the vastly shortened incubation times [49, 56–61]. Secondly, the susceptibility of *tg*a*20* mice to prions and even their prion titers at the terminal stage of disease are similar to those of wild-type mice[49, 62]. Thirdly, both a hemizygous *Prnp* allele and the *tg*a*20* transgene was found to suppress both the granule-cell degeneration of Dpl-overexpressing mice and the chronic demyelinating peripheral neuropathy of *Prnp* ^-/-^ mice[63, 64], indicating that the *tg*a*20* allele is functionally indistinguishable from its wild-type counterpart. The only deviation that was recorded in *tg*a*20* mice is a lack of PrP^C^ expression in cerebellar Purkinje cells[49, 63, 65], possibly due to the absence of a relevant intragenic promoter element (a phenomenon that is not expected to play a role in the studies presented here).

We have found that transcription of the *Prnp* transgene was unaffected by the high-fat diet. This finding is at odds with a previous study using rats subjected to a high-fructose diet [42]. This discrepancy may be due to different diet regimes or to the different animal species. Additionally, the *tg*a*20* allele is controlled by a *Prnp* minigene that does not contain the entirety of the endogenous *Prnp* regulatory sequences, and reverberations of impaired insulin signaling onto the transcription of endogenous *Prnp* may lead to effects undetectable in the experiments described here. However, such a scenario would not apply to modulation of PrP^Sc^ aggregation and/or clearance. Therefore, despite this caveat, our study demonstrates that in contrast to AD or mouse models of AD, insulin resistance does not obviously affect prion pathogenesis. These results indicate that the relationship of insulin resistance between to prions is much blander than that to AD, and are in line with the absence of obvious epidemiological evidence of predisposition to prion disease in T2D patients. Conversely, these data should not be taken to suggest that targeting certain pleiotropic components associated with insulin pathway, such as mTOR and AMPK, would necessarily exert no effects on the pathogenesis of prion diseases.

## Supporting Information

S1 Fig (A) Left: Original uncropped Western blots of p^Ser473^-Akt in ND- or HFD-fed mouse brains. Arrow indicates the specific p^Ser473^-Akt band, unspecific bands are indicated as *. Right: Western blot of Actin for loading control. (B) Left: Original uncropped Western blots of total Akt in ND- or HFD-fed mouse brains. Right: Western blot of Actin for loading control.
(TIF)

S2 Fig (A) Left: Original uncropped Western blots of p^Ser9^-GSK3β in ND- or HFD-fed mouse brains. Arrow indicates the specific p^Ser9^-GSK3β band, unspecific bands are indicated as *. Right: Western blot of Actin for loading control. (B) Left: Original uncropped Western blot of total GSK3β in ND- or HFD-fed mouse brains. Right: Western blot of Actin for loading control.
(TIF)

S1 Table Clinical assessment and scoring of *tga20* mice inoculated with RML6. (DOCX)
