# Supplementary figures and images for: Unaltered Prion Pathogenesis in a Mouse Model of High-Fat Diet-Induced Insulin Resistance
Source: PLoS One. 2015 Dec 14;10(12):e0144983. doi: 10.1371/journal.pone.0144983 (PMC4677814; doi:10.1371/journal.pone.0144983)

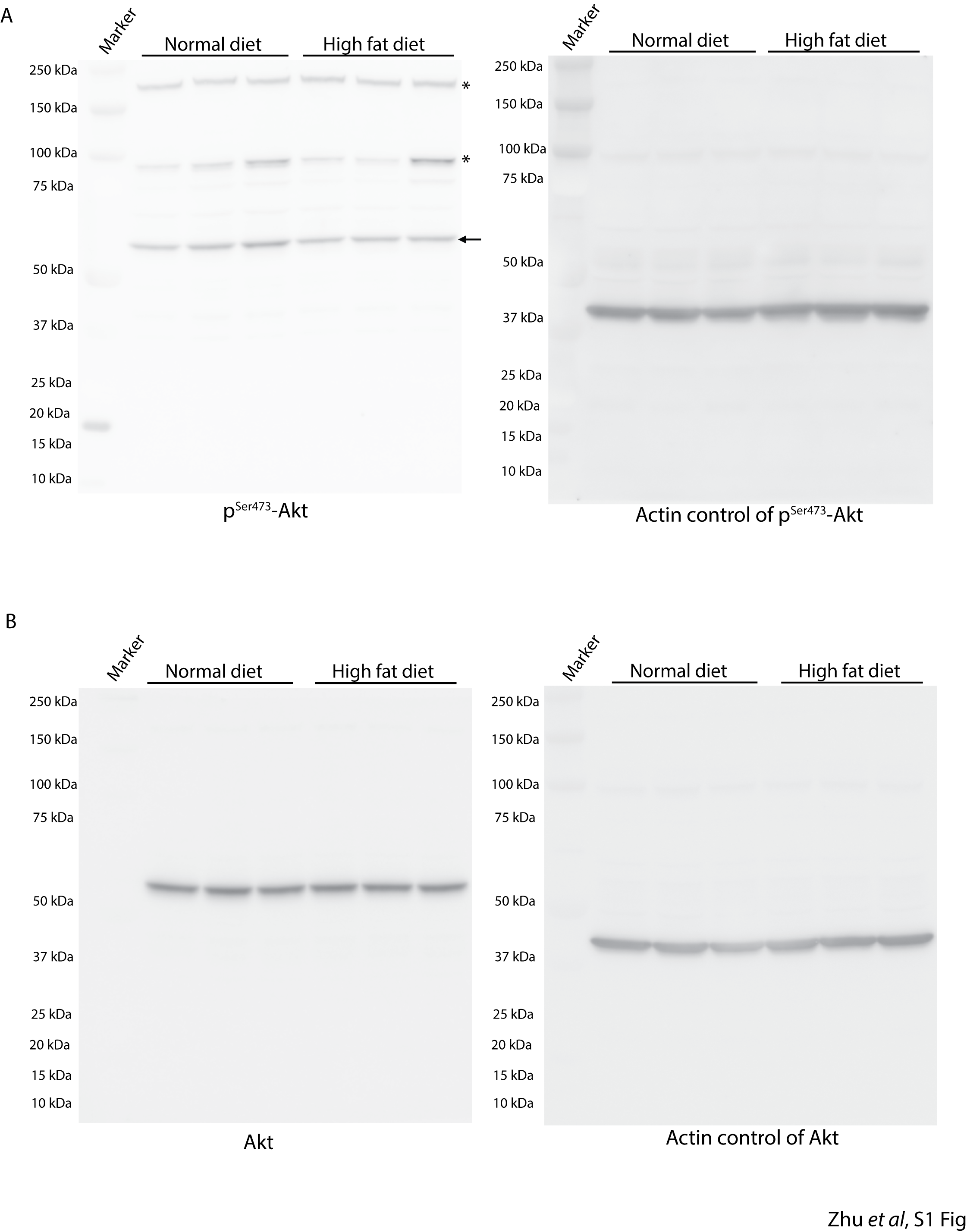

Supplement: S1 Fig — Right: Western blot of Actin for loading control. (TIF) [file pone.0144983.s001.tif]

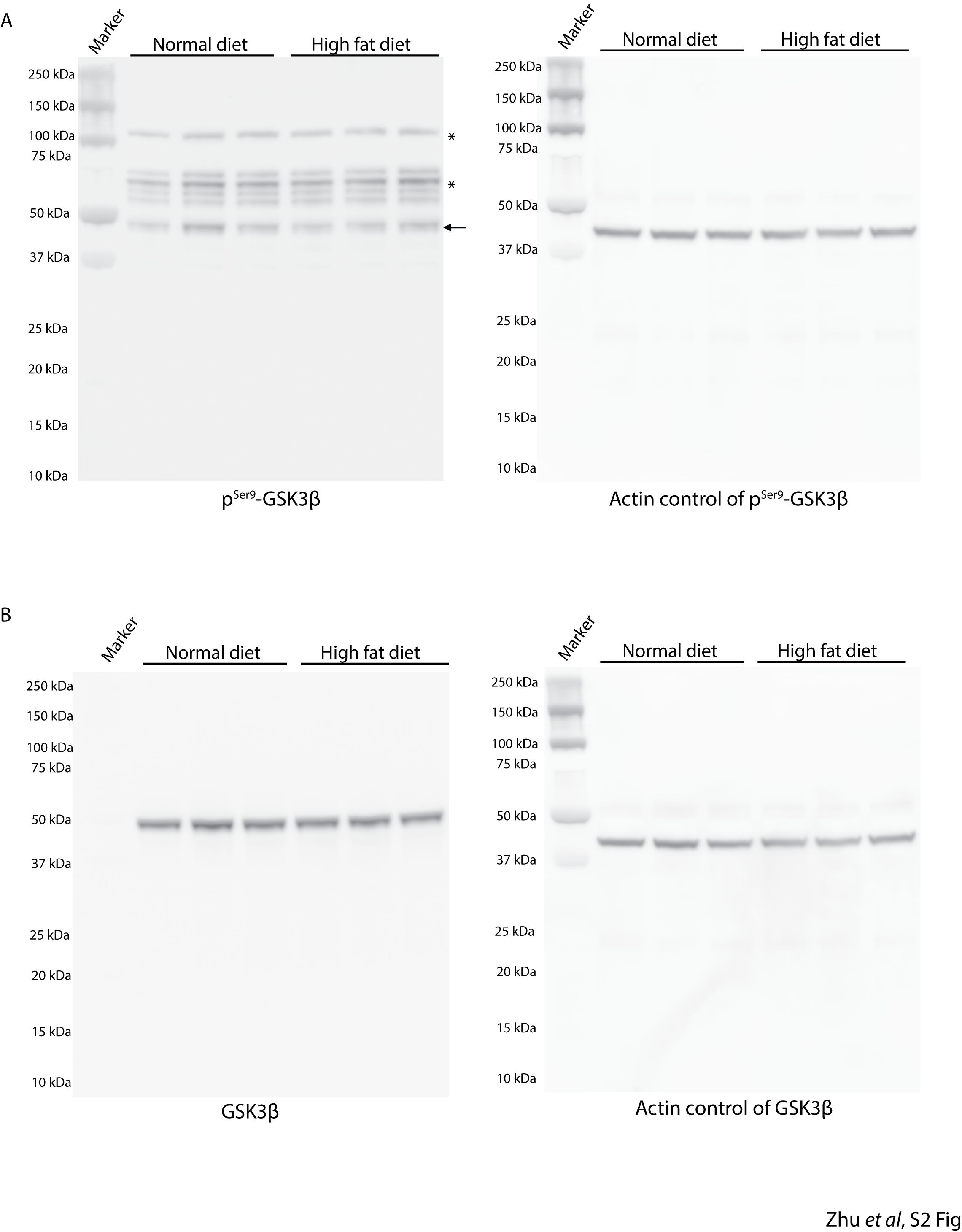

Supplement: S2 Fig — Right: Western blot of Actin for loading control. (TIF) [file pone.0144983.s002.tif]
