# Supplementary material for: Unaltered Prion Pathogenesis in a Mouse Model of High-Fat Diet-Induced Insulin Resistance
Source: PLoS One. 2015 Dec 14;10(12):e0144983. doi: 10.1371/journal.pone.0144983 (PMC4677814; doi:10.1371/journal.pone.0144983)
Supplement: S1 Table — (DOCX) [file pone.0144983.s003.docx]

**S1 table: Clinical assessment and scoring of *tga20* mice inoculated with RML6**

The *tga20* mice were observed every other day after RML6 inoculation for clinical signs including gait, grooming, activity, rough hair coat, limb paresis and ataxia. Once the mice showed the first sign of scrapie (grade 1), they were monitored every day and wet food was supplied in the cage. When the mice reached score grade 2 that hamper the mice reaching water bottle, they were euthanized by CO_2_ inhalation.

| **Score** | **Clinical signs** | **Assessment** | **Action** |
| --- | --- | --- | --- |
| 0 | No detectable signs of abnormal movement |  |  |
| 1 | Waddling gait, mild signs of reduced grooming, rough hair coat, limb weakness, front leg paresis* | Slight rolling while shaking the cage | Provide wet food in the cage;  Observe every day |
| 2 | Ataxia, reduced grooming and activity, paralysis, rolling* | Rolling while shaking the cage | Euthanize immediately if the clinical signs hamper the mice reaching the water bottle |
| 3 | Dead |  |  |

*: observed only in RML6-inoculated tga20 mice.
